# The 4Cs of adaptation tracking: consistency, comparability, comprehensiveness, coherency

**DOI:** 10.1007/s11027-014-9627-7

**Published:** 2015-01-10

**Authors:** James D. Ford, Lea Berrang-Ford

**Affiliations:** Department of Geography, McGill University, Montreal, QC H3A0B9 Canada

**Keywords:** Climate change, Adaptation tracking, Adaptation, Monitoring and evaluation

## Abstract

Adaptation tracking seeks to characterize, monitor, and compare general trends in climate change adaptation over time and across nations. Recognized as essential for evaluating adaptation progress, there have been few attempts to develop systematic approaches for tracking adaptation. This is reflected in polarized opinions, contradictory findings, and lack of understanding on the state of adaptation globally. In this paper, we outline key methodological considerations necessary for adaptation tracking research to produce systematic, rigorous, comparable, and usable insights that can capture the current state of adaptation globally, provide the basis for characterizing and evaluating adaptations taking place, facilitate examination of what conditions explain differences in adaptation action across jurisdictions, and can underpin the monitoring of change in adaptation over time. Specifically, we argue that approaches to adaptation tracking need to (i) utilize a consistent and operational conceptualization of adaptation, (ii) focus on comparable units of analysis, (iii) use and develop comprehensive datasets on adaptation action, and (iv) be coherent with our understanding of what constitutes real adaptation. Collectively, these form the 4Cs of adaptation tracking (consistency, comparability, comprehensiveness, and coherency).

## Introduction

The importance of quantifying and monitoring greenhouse gas emissions is widely recognized, providing measurable outcomes by which the effectiveness of climate policy can be assessed. The significance of the United Nations Framework Convention on Climate Change Kyoto Protocol, for example, has been examined with reference to a decrease in carbon dioxide emissions by 16 % among annex-1 nations between 1990 and 2012 or with respect to global emissions which increased by 52 % over the same period (PBL 2013). Other studies, meanwhile, have examined how various factors affect mitigation action across nations (Tubi et al. 2012; Dolsak 2001, 2009; Baettig and Bernauer 2009). Our ability to similarly evaluate adaptation policy is limited. As chapters in Working Group II (WGII) to the Fifth United Nations Intergovernmental Panel on Climate Change (IPCC) Assessment note, we have only limited and fragmented evidence on adaptation progress globally, reflected in the absence of measurable outcomes or indicators by which adaptation can be evaluated and compared, while our knowledge on what conditions explain differential progress on adaptation across nations, regions, and sectors, is limited (Mimura and Pulwarty 2014; Noble and Huq 2014; Dupuis and Biesbroek 2013a). These deficiencies, in turn, constrain our ability to measure progress: for adaptation, there is no 1990 baseline.

These are significant gaps in the emerging adaptation science, especially given the increased importance of adaptation in climate policy and commitment to funding and adaptation program development by governments at various scales, international institutions, non-governmental organizations (NGOs), and the private sector (Moss et al. 2013; Editorial 2013). Indeed, there is substantial and growing interest in the research and policy community on the need to develop frameworks and indicators for examining adaptation across nations and over time (Ford et al. 2013; Dupuis and Biesbroek 2013a). Despite the need for such work, the literature is lacking the more normative, index-based approaches required at this scale. As Swart et al (2014) argue, most of the research on adaptation focuses on characterizing a small number of cases to examine whether adaptation is occurring and why it is successful or not in particular contexts. This is a necessary and important work, but the insights developed are largely context dependent, with such approaches not well-suited for asking broader level questions about adaptation, including the following: Is adaptation taking place? If so, who is adapting, to what, where, and what types of adaptation are being undertaken? Are we adapting more over time? Which nations, regions, and sectors are leading on adaptation? What elements of adaptive capacity are most significant in determining adaptation action? Why is adaptation progressing in one country or region but not another? What conditions affect adaptation success? (Berrang-Ford et al. 2014; Swart et al. 2014; Preston et al. 2014; Dupuis and Biesbroek 2013a; Berrang-Ford et al. 2015).

Addressing these questions requires studies which seek standardization, generalization, simplification of complexity, and develop broad scale insights. Research methods and techniques used by the adaptation community to-date, however, have generally failed to engage with such approaches, largely eschewing the critical need for breadth as a compliment to research depth. In doing so, adaptation is significantly lagging mitigation in the development of tools, methodologies, and indicators. A number of conceptual, methodological, and institutional challenges have been identified to constrain the development of such work herein: there is wide ranging debate, for instance, on what actually constitutes actual adaptation in general and successful adaptation in particular, few comprehensive datasets on adaptation exist, and the importance of comparative studies on adaptation progress has yet to be fully recognized by the adaptation community and funding agencies (Dupuis and Biesbroek 2013a; Preston et al. 2014; Murtinho and Hayes 2012; Swart et al. 2014). These challenges are not intractable and have been addressed in other areas including global health, international development, and political science.

In this paper, we respond to the need for alternative research approaches in the adaptation field by proposing key components of research design necessary to develop rigorous, systematic, transparent, and ultimately usable insights from which the current status of adaptation across nations and sectors can be characterized, evaluated, and compared. In doing so, the paper builds upon the work of Swart et al (2014) who identify the need for a science of adaptation, by seeking to bring conceptual and methodological clarity to the emerging adaptation tracking work. As such, the paper does not present an assessment tool but outlines the importance of studies being consistent and clear in how adaptation is defined, focusing on comparable units of analysis, using and developing comprehensive datasets on adaptation action, and being coherent with our understanding of what constitutes “real” adaptation.

## The emergence of adaptation tracking research

The last decade has witnessed a rapid increase in adaptation research (Massey and Huitema 2013; Khan and Roberts 2013; Preston et al. 2014), which has recently begun to examine the actual experience of adaptation, assessing the extent and nature of adaptations taking place as well as their success or effectiveness in reducing vulnerability (Ford et al. 2013). The majority of this work focuses on specific policies or programs and has been driven by the needs of development organizations, donors, and governments for measuring the success of supported adaptation initiatives (Villaneuva 2011; Red Cross Red Crescent 2013; Sherman and Ford 2014; Biesbroek et al. 2010; Lamhauge et al. 2013; Brooks et al. 2011b, 2013; Berrang-Ford et al. 2014; Bradley et al. 2014). This research has prioritized developing in-depth, context specific, and primarily qualitative insights on the adaptation process as a basis for evaluating and monitoring intervention/program performance, reflecting the local/regional nature of adaptations of focus in this work. These studies actively seek to work with decision makers to evaluate why and how an adaptation worked, or did not, in a particular context. Methods for achieving this are varied but generally follow process evaluation approaches, where characteristics of adaptation development and implementation are compared to theoretically derived components of adaptation success and best practice (Ford et al. 2013). Evaluation criteria, including effectiveness, efficiency, equity, legitimacy, flexibility, acceptability, mainstreaming, and sustainability, are generally employed in this work to assess adaptations, primarily using qualitative approaches (interviews, focus groups, surveys), although self-reporting metrics such as rating scales, psychometric measures, etc., have also been advocated (de Bruin et al. 2009; Yohe and Tol 2002; Brooks et al. 2011b, 2013; Swim et al. 2011). The strong emphasis on context specificity in this work reflects the widely held, yet increasingly critiqued, perception that adaptation is primarily a local process (Preston et al. 2014).

A much smaller body of scholarship is concerned with the adaptation landscape at regional to global levels, examining if and how adaptation is taking place across nations, how this is changing over time, and identifying predictor’s of adaptation action (Ford et al. 2013; Berrang-Ford et al. 2011; Gagnon-Lebrun and Agrawala 2007; Lesnikowski et al. 2011; Eisenack and Stecker 2012; Krysanova et al. 2010; Massey and Bergsma 2008; Keskitalo 2010; Biesbroek et al. 2010; Reckien et al. 2014; Porter et al. 2014) (Table 1). We term this work “adaptation tracking.” In this nascent field of research, the development and use of indicators is important, providing a systematic and standardized means for evaluating and comparing adaptation over time (i.e., longitudinal assessment) and across regions, countries, and sectors (i.e., case comparison) (Hinkel 2011). The intent of this work is to generalize, quantify, and monitor adaptation for purposes of informing decision makers on the extent to which statements of recognition on adaptation are translating into on-the-ground actions, to learn how different policy contexts are addressing adaptation, to identify and prioritize adaptation needs, to monitor progress on adaptation over time, and to examine factors driving adaptation (Berrang-Ford et al. 2014).

**Table 1:** Examples of approaches to tracking adaptation

Emphasis of the approach	Description	Relevant measures	Sources of information
Progress e.g., Gagnon-Lebrun and Agrawala (2007)	• Emphasis on progress made by governments, NGOs, private sector etc. from articulating adaptation goals to planning and implementation • Views concrete action as more valuable than groundwork	• Have there been vulnerability and impact assessments tailored to the scale, sector, region of focus? • Have different adaptation options been identified? • Have adaptation policies been formulated? • Has adaptation been explicitly incorporated into projects? • Have adaptation measures been implemented? • Has there been learning from past adaptation experience?	• UNFCCC National Communications • UNFCCC Private Sector Initiative • National/regional/sectoral adaptation assessments • Peer reviewed scholarship • Organization websites (e.g., government, civil society organization, health authority) • Legislation • Adaptation databases
Process e.g., Fussel (2008) Mukheibir and Ziervogel (2008)	• Emphasis on procedural aspects of adaptation policy/planning • Views coherent policy-making process to be more likely to produce effective adaptation	• Is there a clear procedural structure in the policy-making process? • Is there evidence of localized impact assessments? • Is there evidence of building M&E into the adaptation process? • Is there evidence of inclusion of key stakeholders? • Have adaptation concerns been prioritized in the policy-making domain? • Has adaptation been incorporated into the development process? • Has adaptation been incorporated into Disaster Risk Reduction programs? • Is there a prioritization among adaptation policies • How are uncertainties being managed?	• Adaptation planning documents Adaptation program descriptions • Consultation documents • Boundary organizations • Institutional structure analysis • Adaptation readiness evaluations • Program development • Decision maker surveys
Diversity e.g., Carmin et al (2012) Lesnikowski et al (2011; 2014)	• Emphasis on the need to tackle vulnerability across sectors • Values variety of adaptation policies • Values diversity of impacts and sectors addressed • Highlights the importance of diverse typologies to address different problems	• How many/which impacts are being addressed? • How many/which sectors are being strengthened? • Which policy typologies are being used (e.g., direct management vs. soft and open policies)	• Government policy summaries • Sectoral adaptation reports (e.g., transportation ministries, utilities ministries, port authorities) • Global/regional/local surveys of adaptation activity • UNFCCC National Communications • IPCC Assessment Reports
Quality e.g., Dupuis and Biesbroek (2013)	• Evaluates the success of policy on increasing resilience Emphasis on purposeful and substantive aspects of adaptation policy • Outcome oriented, examining the quality of existing adaptation policies	• Is the policy explicitly designed to manage the impacts of climate change? • Does the policy reduce climate change vulnerability?	• Documents monitoring implementation • Independent program evaluation • NGO/private sector assessments • Public and private policy analyses • Peer reviewed scholarship

The emergence of adaptation tracking research reflects a number of factors. Firstly, as the adaptation field has expanded, there has been increasing frustration that investments in adaptation research have not translated into action, with a number of recent articles noting that this stems from the underlying, untested heuristics framing much adaptation work, and dominance of practice orientated case study methodologies to the exclusion of other approaches (Swart et al. 2014; Berrang-Ford et al. 2014; Preston et al. 2014; Massey et al. 2014; Burton and Mustelin 2013; Bassett and Fogelman 2013). Herein, adaptation tracking studies are recognized as essential for theorizing and testing fundamental assumptions about adaptation based on cross-national comparative analysis of how different contexts and jurisdictions approach adaptation, and for learning what determines success of policy intervention (Dupuis and Biesbroek 2013a). Fundamental questions of importance here would include examining what components of adaptive capacity are most important for determining successful adaptation, how they operate in different contexts, and what factors operate as effect modifiers (Berrang-Ford et al. 2014). While such arguments for the importance of adaptation tracking have been primarily articulated in an academic setting, the questions raised are essential for developing a comprehensive evidence base on what works in an adaptation context.

Secondly, the adaptation tracking field is emerging in response to the needs of national governments, international organizations, and the scientific community, with NGOs and the private sector also identifying interest (Sovacool et al. 2012; Lesnikowski et al. 2014; Brooks et al. 2013; Editorial 2013; Lamhauge et al. 2013; Biagini et al. 2014). This reflects a number of factors—summarized in Table 2—including the need to evaluate whether adaptation support is translating into actions, identify future priorities, ensure resources are being invested in areas with the greatest need, and inform governance systems on the current status and gaps in adaptation action (Ford et al. 2013). As adaptation funds have begun to be disbursed through the United Nations Framework Convention on Climate Change (UNFCCC), for example, Parties, NGOs, and United Nations (UN) bodies have expressed the need to examine the success of funds invested for accountability purposes and to ensure resources are being effectively utilized, with the Cancun Agreement explicitly recognizing the need to monitor and review adaptation across nations. Governments at various levels have also expressed interest in measuring progress towards meeting the objectives of national adaptation strategies, learning how other jurisdictions are adapting, and evaluating progress over time (Lesnikowski et al. 2014).

**Table 2:** Potential users of adaptation tracking studies and the questions that can be answered

Potential users of adaptation tracking research	Questions adaptation tracking research can help answer
International organizations that fund adaptation (e.g., World Bank, regional development banks, UN organizations)	- Are adaptation programs stimulating action on the ground (e.g., GEF programs)? - Which nations have the greatest need for adaptation support? - Are actions consistent with the risks posed by climate change? - How is adaptation changing over time?
UNFCCC (Cancun Agreement Decision 1, paragraphs 14 and 20 explicitly recognizes need to monitor and review adaptation)	- Are Nations meeting their responsibilities to adaptation as set out in the UNFCCC? - How can adaptation funds be most effectively invested? - In what areas and regions is technology and knowledge transfer for adaptation needed? - Are we progressing on adaptation?
Government (various scales: national, regional, municipal)	- How does performance compare to other governments? - Are there transferable lessons from other governments? - Is progress being made to meet adaptation planning objectives? - Where are the gaps in adaptation? - Are projected risks being addressed?
Research community	- Is the adaptation response consistent with the risks posed? - What factors explain adaptation progress and do they vary across region, nation, sector? - Which nations are leaders in adaptation and what lessons do they hold for promoting adaptation globally?
NGOs	- Which nations and what sectors need adaptation support? - Is the international response to adaptation consistent with the risks posed and is it progressing?
Private firms/consultancy	- What are available methods to measure adaptation progress? - What types of adaptation initiatives currently exist elsewhere and can be transferred? - How can the policy process be changed to induce more effective adaptation?

For purposes of tracking progress, adaptation is a different problem from mitigation, which can be assessed vis-à-vis greenhouse gas emissions. The ultimate goal of adaptation is to reduce harm to future climate change and, theoretically, could be assessed with reference to avoided future impacts, where loss metrics (e.g., morbidity, mortality, economic loss attributable to climate) could be monitored to evaluate progress towards a more adaptable society. The use of such metrics, however, depends on avoided impacts being observable, measureable, and attributable to adaptation, thus limiting the applicability of outcome indicators given conceptual and methodological challenges (Adger et al. 2005; Brooks et al. 2011a; Ford et al. 2013). Proxies are therefore needed for developing a baseline on the current status of adaptation and for measuring progress (Table 3). The majority of studies use reporting on adaptation policies, programs, and initiatives as a proxy on the extent to which adaptation is taking place and for examining potential effectiveness in reducing vulnerability (e.g., adaptation databases, UNFCCC National Communications, peer reviewed and grey literature, national adaptation assessments, adaptation planning documents etc.) (Table 3). While such reporting is an imperfect proxy subject to reporting bias, challenges associated with implementation deficit, and varying level of detail provided (Dupuis and Knoepfel 2013b; Hupe et al. 2014), there are few alternative data sources available across nations for tracking purposes that provide the level of detail necessary (Bizikova et al. 2015; Sud et al. 2015; Gagnon-Lebrun and Agrawala 2007). Moreover, such reporting has been used for comparable policy tracking for global health and social policy to identify and monitor general policy trends, locate leaders and laggards, and examine change over time (Ford et al. 2013; Earle et al. 2011; Heymann et al. 2011; Heymann and McNeill 2013).

**Table 3:** Data sources used in adaptation tracking research

Data sources	Context of use	Strengths	Limitations
National Communications to the UNFCCC	- Examine status of adaptation in annex 1 nations (Lesnikowski et al. 2011; Gagnon-Lebrun and Agrawala 2007) and globally (Lesnikowski et al. 2013) (creation of adaptation index) - Identify adaptation predictors globally (Lesnikowski et al. 2014)	- Standardized, systematic, transparent data collection - Regular reporting for annex-1 nations - National-level data globally - Accessible online in one location	- Not available for all nations - Primarily mitigation focused, limited detail on adaptation - Reporting bias - National focus
Published climate initiatives	- Assess climate preparedness in UK urban areas (Heidrich et al. 2013; Reckien et al. 2014) (creation of climate preparedness index)	- Detailed information on adaptation initiatives and programs - Widely available documents (in a high income context)	- Lack of standardization in reporting - Discrepancies in reports - Resource intensive: requires the identification, retrieval, and collation of documents
Website content	- Document civil society action on adaptation with regards health in Canada (Poutiainen et al. 2013) - Identify community based adaptation actions in Africa (Mannke 2011) - Identify OECD actions to prepare for impacts of climate change on infectious disease (Panic and Ford 2013)	- Detailed information on adaptation initiatives and programs - Diversity of adaptations reported and captured - Diversity of reporting scales - On-the-ground adaptation reporting	- Outdated content - Identification, retrieval and collation of information challenges - Lack of standardization - Reporting bias based on technological capacity - Varying detail on adaptation
UNFCCC Private Sector Initiative	- Scoping of the current state of adaptation in the private sector (Surminski 2013)	- Standardized reporting template - Information on private sector	- Limited coverage - Reporting bias - Limited detail on actions
Peer reviewed journal articles	- Characterize the nature and extent of adaptation globally (Berrang-Ford et al. 2011), in annex-1 nations (Ford et al. 2011), in high risks areas including the Arctic and mountain regions (Ford et al. 2014; McDowell et al. 2014), among households in the UK (Porter et al. 2014)	- Easily accessible, rapid assessment - High quality reporting from varying scales	- Reporting bias - Lack of standardization - Varying detail on adaptation
National Adaptation Strategies	- Evaluation of national level adaptation in the EU (Biesbroek et al. 2010; Massey and Bergsma 2008)	- Comparable - Standardized and systematic - National-level data	- National focus - Reporting bias to countries with high capacity - Data exists for European countries exclusively
Peer reviewed and grey literature	- Survey on the state of adaptation in the UK (Tompkins et al. 2010) - Survey on the state of adaptation in arid and semi-arid regions (Ford et al. 2014; Sud et al 2015; Bizikova et al 2015)	- Depth of information and diversity of adaptations captured - Diversity of conceptual frameworks	- Time requirements - Lack of standardization - Varying focus, detail, and quality
Legislation	- Number of laws with adaptation focus (Townshend et al. 2013)	- Broad scope - National-level data available globally	- Legislative approach not taken in all countries - Institutional contexts vary by nation - Formal laws not necessarily indicative of action
Surveys with policy makers	- Survey of elite policy makers in 36 EU nations to examine development of national level adaptation policies and practices (Massey et al. 2014)	- Document current state of action on adaptation - Standardization - Not limited by what is reported in documents - Depth of insights	- Challenge of getting sufficient response rate within and across nations - Time intensive

## Methodological considerations for adaptation tracking

The emerging adaptation tracking subfield has developed a baseline understanding of adaptation in specific contexts, piloting different approaches, and methods. Yet the polarized opinion and contradictory findings on the current state of adaptation are indicative of a weak understanding of what adaptation and adaptation progress means (Noble and Huq 2014; Dupuis and Biesbroek 2013a) (Table 1), while there is little agreement or standardization on how adaptation reporting should be used for adaptation tracking purposes. This in part reflects the complexity of adaptation, although the conceptual and methodological challenge of tracking adaptation is not unique to climate policy, with a comparable scholarship focusing on problems of similar scope (e.g., global health, social policy). To inform the development of approaches for adaptation tracking, we draw upon this literature and emerging work in an adaptation context to explicitly outline key methodological considerations necessary for global adaptation tracking research to produce systematic, rigorous, comparable, and usable insights that can (i) capture the current state of adaptation across nations, (ii) provide the basis for characterizing and evaluating adaptations taking place in different settings, and (iii) underpin the monitoring of change in adaptation over time.

**Fig. 1:**
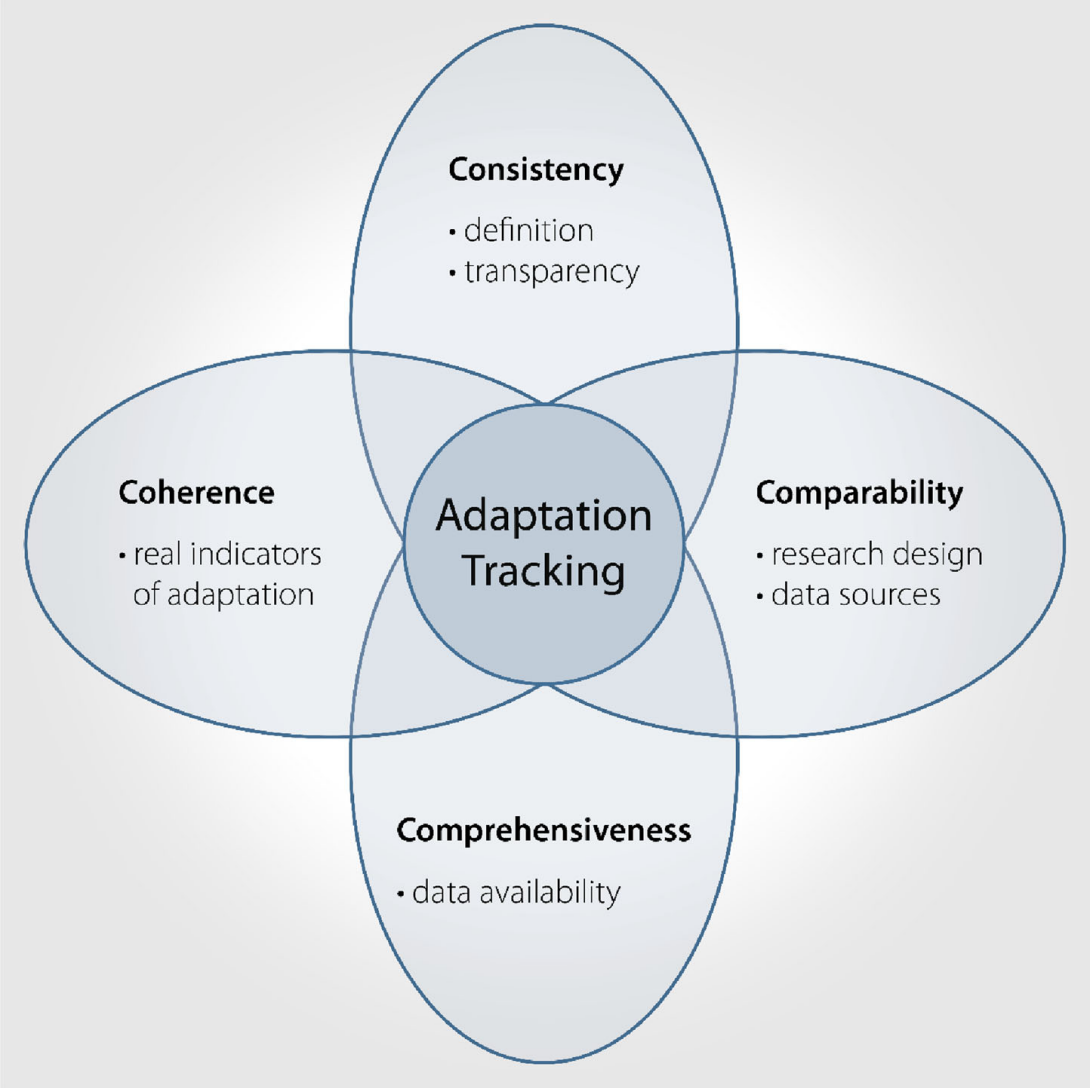
The 4Cs of adaptation tracking

Borrowing from systematic data collection approaches in global health, and based on the foundation of empirical study design, we collectively term these the 4Cs of adaptation tracking (Fig. 1). In doing so, we are explicitly drawing on two key contributions within public health from which we seek to translate lessons for adaptation research. Firstly, the field of public health has, and continues to be, a leader in the development and application of methods for systematic approaches to literature review, seeking transparent and explicit methods for evidence synthesis (Berrang-Ford et al. 2015). Though traditionally confined to health and health-related fields, systematic approaches to evidence synthesis provide significant opportunity for grappling with diverse evidence of climate change adaptation, and we have herein seen recent emergence in the use of systematic review approaches within the adaptation scholarship (Hardee and Mutunga 2010; Murtinho and Hayes 2012; Biesbroek et al. 2013; Kamau and Mwaura 2013). Second, in the 1980s, public health scholars collaboratively developed an integrated index to track health morbidity and mortality broadly across time and space, leading to the creation of the disability adjusted life year (DALY) and the first Global Burden of Disease Study (GBDS) in 1990 (Murray et al. 1994). A seemingly insurmountable challenge, and highly controversial at the time, these efforts have contributed substantively to systematic tracking of global health, and stimulated a new discipline in health metrics. Though global in focus, the GBDS has motivated broader methodological innovation in measuring health impact, including more localized metrics for tracking health burden. We see important parallels here with climate adaptation and seek to stimulate a similar move towards methodological development of innovative approaches—though not unified metrics like the DALY—for tracking global adaptation. Underpinning the GBDS was the goal of seeking comprehensive, consistent, and comparable methods for evaluating health burden globally. We thus draw conceptually from the GBDS here, proposing the 4Cs of adaptation tracking.

Each of the Cs is central to adaptation tracking, regardless of whether the primary aim is comparing adaptation progress across cases or longitudinal tracking. Where possible, we use examples to illustrate application of the Cs, noting that there are no studies as yet and to our knowledge, that perform well across all components. Indeed, integrating all the 4Cs into research design presents a significant challenge for the adaptation tracking community. We use an example for our work in Table 4 to demonstrate the application of the 4Cs.

**Table 4:** Application of methodological considerations for tracking adaptation in large urban areas globally (Araos et al. In review)

Methodological consideration	Application	Challenges
Consistency	• Step 1: Adaptations defined broadly as “adjustments in natural or human systems in response to actual or expected climate stimuli and their effects.” • Step 2: Record adaptation initiatives only if they are explicitly communicated as adaptations to climate change. • Step 3: Organize adaptation policies into a database of discrete initiatives.	• Some initiatives may reduce vulnerability but not be framed as climate change adaptation. • Some initiatives address natural climate variability rather than long term change. • Some initiatives may be maladaptive.
Comparability	• Urban municipal governments defined as the unit of comparison. • Adaptation initiatives recorded only if they are undertaken by the municipal government. • Large cities analyzed (>1 m), small cities excluded. • Systematic web search for Adaptation Plans, Climate Action Plans, NGO-partnered initiatives, and official government websites.	• Exclusion of other actors undertaking adaptation: - Exclude adaptation by private individuals or households. - Exclude adaptation from the private sector. - Exclude adaptation from other governmental scales (national / regional). • Lack of generalizable metrics to evaluate effectiveness of adaptation.
Comprehensiveness	• Use translators to capture >90 % of cities over 1 m. • Classify initiatives sectorally to grasp breadth of adaptation (e.g., water supply, transportation, human health). • Analyze 402 cities to produce a large enough dataset for inferential statistical analysis: - Identify and analyze drivers of adaptation (e.g., GDP, population, good governance index).	• Reporting bias: - Measuring the ability to communicate adaptation rather than adaptation itself. - Low capacity governments may not publish adaptation projects, but may be partnered with other organizations to undertake initiatives. • Logistical and resources constraints in analyzing large number of cities with diverse languages.
Coherence	• Use policy classification methods coherent with existing theory: - Groundwork vs. action. - Which vulnerabilities are addressed? (e.g., temperature increase, soil erosion, sea level rise). - Which sectors are targeted? (e.g., energy supply, infrastructure, social services). - What is the policy’s typology? (e.g., management, capacity building, financing, research). • Develop methods to capture substantiality of the initiatives. • Match existing and planned initiatives against stated commitments and goals. • Perform qualitative case studies to identify policy pathways facilitating adaptation	• Conceptual difficulty in measuring the impact of adaptation policy – how do we measure averted risk? • Variations in the definition of adaptation “success”. • Fuzziness of adaptation goals across government scales. • Difficulty in sorting policies intentionally designed as adaptation to climate change vs. re-labeled existing policies.

This table illustrates the application of the 4Cs in the context of a project tracking adaptation in urban areas globally. The project analyzed 402 urban municipal governments and classified cities according to their adaptation profiles. We used systematic web searches to identify government adaptation documents, and then extracted discrete adaptation initiatives into a database. We only gathered initiatives if they were explicitly communicated as adaptations to climate change. We retrieved adaptation data only for cities over one million inhabitants and from cities in which the official languages was spoken by at least five cities total. Once gather adaptation data we classified initiatives based on whether they were groundwork or action, which impacts and sectors they targeted, and adaptation policy typology

### Consistency

If progress on adaptation is to be monitored over time and compared across nations, a consistent and operational conceptualization of adaptation is needed so that any documented differences or change are not a function of definitional inconsistency. The commonly used IPCC (2007) definition of adaptation as “adjustments in natural or human systems in response to actual or expected climate stimuli and their effects,” lacks specificity for tracking, where the first task is to identify what actually counts as adaptation. This can be challenging, with adjustments potentially taking a myriad of forms and functions, and may involve specific responses to a known risk or seek to enhance overall capacity to adapt, can be autonomous or planned, focus on reducing present days risks or have a future focus, and may be completely or only partially motivated by climate change (Smit et al. 1999, 2000; Smithers and Smit 1997; Noble and Huq 2014). Perspectives on what is adaptation thus differ widely, determining the extent to which studies are able to find evidence of adaptation taking place; this fuzziness in the scope and boundaries of adaptation has been termed the dependent variable problem (Dupuis and Biesbroek 2013a).

The challenge of specifying what counts as adaptation poses a significant problem for adaptation tracking. Expecting all adaptation studies to use a common conceptualization of adaptation is unrealistic, however, evidenced in the context of similar challenges facing efforts to measure vulnerability (Hinkel 2011; Klein 2009; Klein and Moehner 2011). Indeed, the plurality of definitional starting points can bring diverse insights to measuring adaptation progress. Notwithstanding, tracking studies need to be internally consistent in their definition of adaptation if comparing over space and time, transparent in defining inclusion and exclusion criteria for what they consider as adaptation to allow for replication of the study by other research teams, and clearly acknowledge the limits implied by the definition used.

Two alternate conceptualizations of adaptation offer a starting point for tracking studies. A narrow view of adaptation would focus on identifying, characterizing, and monitoring purposefully designed responses to address climate change impacts that contribute to reducing vulnerability and/or taking advantage of new opportunities (Dupuis and Biesbroek 2013a). This follows from arguments that climate change poses unique risks which require specific policies to address future vulnerabilities (Adger and Barnett 2009; O’Brien 2012; Smith et al. 2011; Eakin et al. 2014). A broad view of adaptation would consider policies designed to generally reduce risk in which climate change may be one of multiple rationales for adaptation, including statements of recognition on the need for developing response options and groundwork action to inform and prepare for adaptation (Tompkins et al. 2010). Such an approach reflects recognition that adaptation is a process of multiple stages, may be most effective when mainstreamed into ongoing policy priorities, and involves addressing the broader socioeconomic determinants of climate vulnerability (Dovers 2009). Both perspectives offer diverse and complimentary perspectives on the state of adaptation and also entail risks. The broad definition risks capturing symbolic policies with limited impact on vulnerability; the narrow definition may fail to capture important capacity building activities essential for vulnerability reduction. For this reason, and to minimize bias that relying on single definitions may bring, a diversity of definitional starting points are essential to bring multiple lenses from which to view adaptation progress.

The importance of consistency considerations in research design has not been widely addressed in the scholarship, with the majority of tracking studies providing limited operational detail beyond basic definitions of adaptation, limiting the ability for comparative analysis, for monitoring progress over time, and for study replication. Furthermore, studies typically identify policies/programs as adaptations if explicitly identified as such in the data source being used, but this creates challenges for consistency given the often limited detail given on how and for what purposes an adaptation was originally defined as such. Particularly, if monitoring change in adaptation over time, but also for cross case comparison, what is promoted as “adaptation” may differ widely depending on current scientific and policy norms and political factors. Exceptions include Lesnikowski et al. (2011, 2013) in their work documenting adaptation in the health sector, who outline detailed and explicit criteria by which policy responses are classified as adaptations, with distinction made between: statements of recognition which constitute the most basic demonstration that countries have identified climate change as a problem; groundwork actions which are considered first steps necessary to inform and prepare for adaptation, but do not explicitly indicate tangible changes in policy or delivery of government services (e.g., vulnerability assessments, research on adaptation options, conceptual tools, stakeholder and networking opportunities); and adaptation actions which refer to tangible changes in response to predicted or experienced impacts of climate change. Similarly, at a conceptual level, Dupuis and Biesbroek (2013a) propose a typology by which adaptation can be identified and characterized by the policy’s intentionality and substantiality.

### Comparability

A fundamental component of systematically tracking adaptation is that methods are guided by empirical sampling techniques. This necessitates a comparable unit of analysis: who or what, exactly, is being compared? Existing literature provides a range of case studies of adaptation from cities, countries, regions, and institutions. Comparing city-level to country-level adaptation initiatives (unless explicitly focusing on cross-jurisdictional patterns), however, is conceptually equivalent to comparing apples and oranges. To measure progress, adaptation tracking initiatives must define a spatial and temporal unit of analysis, or denominator, from which adaptation metrics can be reasonably compared. This might involve evaluating national-level adaptation progress among nations over the past 5 years or comparison of municipal adaptation programming for a defined period of time. Efforts to track adaptation have focused on particular sectors (e.g., health, private sector), regions (e.g., high-income nations, continents, high-risks nations, urban areas, mountain regions), and institutions (Heidrich et al. 2013; Mannke 2011; Lesnikowski et al. 2011, 2013; Surminski 2013; Tompkins et al. 2010; Gagnon-Lebrun and Agrawala 2007; Keskitalo 2010; Poutiainen et al. 2013; Sovacool et al. 2012; Reckien et al. 2014; Ford et al. 2014; McDowell et al. 2014). This is invariably complicated where there are differing jurisdictions—comparing adaptation among global cities where some cities have greater devolved power for adaptation programming than others, for example. Similarly, comparing adaptation across nations is complicated by different jurisdictional structures and sizes: the Canadian federal mandate, for example, is more directed towards assisting lower level jurisdictions by providing information, resources, and guidance, compared to many European nations where national governments have a much strong role in supporting actual adaptation actions (Dickinson and Burton 2011; Isoard 2011).

Similarly, selection of data sources for adaptation tracking should be guided by empirical sampling approaches to ensure a representative sample from which to infer trends in adaptation over time. While there is likely much literature and data on adaptation among leading nations, cities, and institutions from which adaptation can be evaluated, if we are interested in general adaptation progress, then we must also include laggards in our datasets. Herein, reporting bias will continue to be a challenge for adaptation tracking: does a lack of publically available data or information on adaptation activities reflect poor adaptation progress or simply poor reporting? More standardized guidelines for collection of adaptation indicators with universal and consistent reporting would dramatically enhance access to comparable adaptation datasets at the national level and limit the impacts of reporting bias (Lesnikowski et al. 2014).

While more comparable datasets guided by efforts to systematically and empirically compare adaptation are emerging, there is negligible focus on adaptation progress over time. Adaptation tracking should therein aim not only to compare between units of analysis (e.g., nations) but also monitor change and progress. This requires longitudinal data reporting indicators of adaptation on a yearly or periodic basis (e.g., every 5 years). A snapshot of greenhouse gas emissions for a single year would be considered unsatisfactory in the context of tracking and monitoring mitigation, and the same must apply to adaptation. While no adaptation baseline exists, adaptation can at minimum be conceptualized through evaluation of progress and establishment of identifiable and comparable milestones.

An important component of comparability is also transparency in the methodology used for adaptation tracking. This is necessary to underpin longitudinal analysis of adaptation progress and comparison across cases, to facilitate use of baseline data by other research groups, to ensure consistency, and to allow independent replication of results.

### Comprehensiveness

Our ability to infer generalizable trends and patterns and compare across nations or regions necessitates datasets large enough and with enough detail to capture a range of adaptation experiences, outcomes, and progress. Comprehensiveness herein reflects the extent to which data are available for a large number of countries, regions, or other units of analysis, and, for purposes of longitudinal assessment, are updated over time. While qualitative research will continue to play a critical role in exploring the depth of adaptation processes in specific contexts, there is significant value in developing datasets sufficiently comprehensive to allow quantitative analysis and an exploration of the breadth of adaptation progress across nations, using systematic approaches and comparable, consistent indicators. This presents challenges given that standardized and comprehensive data sources for an adaptation context are often unavailable (Table 3). Lesnikowski et al. (2014), for example, in their global analysis of national-level adaptation based on reporting in UNFCCC National Communications, were only able to focus on 117/195 nations due to a lack of recent reporting by many low- and middle-income countries (including large nations such as China). This reflects the risk that information on adaptation may be least available from global regions with the greatest vulnerability and need for adaptation.

As noted above for comparability, the critical factor in ensuring comprehensiveness is that adaptation datasets are, to the greatest degree possible, developed and evaluated using principles of empirical sampling. This implies not only that we have comparable data for countries (or other unit of analysis) in our dataset but also that we have as complete—and representative—a dataset as possible if we want to infer results for broader insight. Research focused on adaptation challenges and progress in developed nations certainly contributes to our understanding of adaptation, but there is an implicit bias towards the low hanging fruit regions or topics for which data are more available and voluminous. There is, for example, a research gap in our understanding of adaptation action and progress in middle income nations (Berrang-Ford et al. 2011), few datasets of adaptation in global cities which include a large and representative number of low-income cities (Araos et al., in review), and the great majority of adaptation data sources which include only a sample of regions or jurisdictions among those to which we would like to infer. The National Communications (NCs) to the UNFCCC are excellent and relatively comparable sources of adaptation information yet are largely available on a longitudinal basis only among higher-income nations. Adaptation information in the National Adaptation Programmes of Action (NAPAs) in contrast is ad hoc, only sporadically updated, and often aspirational. The potential for the NCs to provide a systematic database of global adaptation progress is thus constrained by the absence of comprehensive inclusion of all—or at least a representative sample of—nations. A more strategic approach to systematic adaptation tracking should seek to identify gaps in information or coverage and intentionally collect data for these regions or areas.

There are two options to seeking more comprehensive adaptation data sources. First, we must aspire to explicitly integrate comprehensiveness as a sampling strategy when developing new data sources. Here again, more standardized national reporting (e.g., through the UNFCCC) that focuses on adaptation indicators would substantially enhance our ability to assemble larger datasets to track global and regional adaptation more systematically. Organizations and researchers seeking to systematically track adaptation should ask: To what extent are the observations in this dataset internally and externally valid for making broader inferences? Who or what is excluded from this dataset, and to what extent might this affect the nature or generalizability of results to inform broader insight? Secondly, we should seek innovative data sources that provide more comprehensive access to adaptation information that is not readily available through standard reporting structures. Surveying policy makers and practitioners is one potential alternative approach to relying on publically available information, and Massey et al. (2014) survey elite policy makers to document adaptation policies and programs taking place in 36 European nations. The difficulty of getting sufficient response rates within and across nations to develop necessary insights, however, was identified as a major challenge in this work, a challenge likely to be compounded for global scale studies where multiple languages need to be spoken (noting Google translate can often be effectively used for information online). Another alternative would be to leverage new opportunities via the rapidly evolving Web 2.0, including automated web scraping, crowd sourcing, and data mining approaches. These tools remain relatively unexplored and undeveloped yet present potentially new avenues to track adaptation perceptions and activities outside of formal governmental sectors and governance structures.

### Coherence

A great challenge for adaptation tracking is developing measureable indicators that reflect substantive adaptation. Methods should thus aim to be coherent with our existing understanding of what constitutes real adaptation. For purposes of measuring adaptation progress, for example, it is important that tracking approaches go beyond documenting the number of adaptations, widely used as a basis for measuring progress, to also capture the substance of policy development: the quantity of adaptations observed is not necessarily indicative of progress towards a more adaptable society, and adaptation efforts may be either maladaptive or merely labeled as adaptation without substantive impact (Knill et al. 2012; Ford et al. 2013; Hupe et al. 2014; Dupuis and Biesbroek 2013a; Massey et al. 2014). Such aspirations for indicators that reflect deeper and more critical aspects of adaptation success may be difficult to find in practice and impossible to access in a comparable and comprehensive beyond disparate case studies, however. There is a risk, therefore, that in seeking comparable and comprehensive adaptation data, we unintentionally water down the quality (coherence) of our measures.

This methodological challenge should not prevent adaptation tracking efforts from seeking to find measureable indicators that reflect substantive aspects of adaptation coherent with qualitative and theoretical research. One such approach could involve examining the adequacy of documented policies and programs against identified adaptation commitments, goals, and needs and has been used to compare and monitor responses across UN nations on various components of social policy (e.g., labor conditions, poverty alleviation) (Earle et al. 2011; Heymann et al. 2011; Heymann and McNeill 2013). In an adaptation context, this could involve evaluating adaptations against the types of risks addressed and relevance vis-à-vis projected changes, targeting of vulnerable populations, stage of intervention, and extent to which future risks are considered, or involve evaluating if adaptations are targeting governance structures and processes that determine the presumed ability of nations to adapt (i.e., adaptation readiness) (Ford et al. 2013). Lesnikowski et al. (2013), for example, develop an adaptation index based on stage of adaptation reported in UNFCCC National Communications (statement of recognition, groundwork, action), while Heidrich et al. (2013) develop a climate change preparedness score based on breadth of measures reported using documentation on climate policy in urban areas in the UK. Such indices can be used to track adaptation over time and across nations and can underpin analysis of drivers of adaptation action (Berrang-Ford et al. 2014).

Dupuis and Biesbroek (2013a), however, argue that more theoretically informed indicators of progress are needed that more substantially capture effectiveness alongside actions and propose a proximity-to-target approach where documented adaptations can be compared to a theoretically derived model of successful adaptation. Here, adaptation is conceptualized by intentionality, capturing the extent to which policies are purposefully designed or changed to manage the impacts of climate change and reduce vulnerability, and substantiality, capturing the extent to which a policy contributes to actually reducing vulnerability or benefiting from climate change opportunities. To be meaningful and coherent with adaptation research, adaptation tracking should seek to be guided by theory and identify thoughtful proxies of adaptation progress. To this end, achieving coherence of indicators with our theoretical understanding of what constitutes real adaptation may be unfeasible in practice. There is an inevitable trade-off between the breadth of comparable and comprehensive datasets and the substantive depth (coherence) sought to investigate adaptation progress that goes beyond simple metrics and crude indicators of adaptation.

## Conclusion

Over the last decade, adaptation has emerged as a central component of climate policy, increasingly prioritized in government policy across scales, by NGOs, international institutions, and the private sector. Our understanding on the current state of adaptation globally, however, remains limited to snapshots provided by global/national assessments and case studies from different regions. An emerging adaptation tracking subfield has also begun to develop and has been influential in creating a baseline understanding of adaptation across regions, nations, and sectors, piloting different approaches and methods. Yet discrepancies and inconsistencies in work that has been completed are indicative of the immaturity of the adaptation tracking field. With the creation of new funding streams and programs for adaptation at international and national levels, there is a need for systematic, rigorous, and transparent approaches to adaptation tracking research focused on developing indicators by which the current state of adaptation can be characterized, monitored, and compared.

In this paper, we propose key components of research design that should be used to guide adaptation tracking studies. The work advances further a nascent scholarship assessing adaptation progress, specifically focusing on components of research design necessary for longitudinal analysis, comparison across nations, and the development of adaptation indicators. Herein, indicators provide a systematic and standardized means for characterizing the state of adaptation at a specific point in time and from which future progress can be monitored, evaluated, and communicated. In a tracking context, indicators can provide a direct measure of adaptations taking place, and contrast to vulnerability where the use of indicators to characterize and monitor trends has been widely critiqued, reflecting the nature of vulnerability as a potential state of affairs and lack of agreement on determinants of vulnerability (Hinkel 2011; Barnett et al. 2008; Klein 2009). It has been argued in the general scholarship, however, that indicators may mislead policy, directing attention to interventions that can be measured and focusing on improving rankings as opposed to developing policies that are effective in actually addressing a problem. The development of indicators also often involves trading breadth for depth, thus limiting the ability to capture whether effective adaptations are being implemented, while questions of equity and power arise around who defines adaptation. For these reasons, we emphasize that developing adaptation indicators for global scale tracking purposes must occur in parallel with qualitative studies examining adaptations in specific places; together, both type of study can make a powerful contribution towards informing adaptation policy priorities. The need for such diversity in methodological approaches for adaptation has not yet been fully articulated, with the field remaining dominated by context specific studies (Swart et al. 2014; Preston et al. 2014).

Systematic tracking also requires standardized reporting on adaptation. Mitigation reporting already consists of well-developed methodologies for inventorying emissions, yet only a limited number of data sources collect information on adaptation in a rigorous, consistent, transparent, timely, and comprehensive manner. National Communications (NCs) to the UNFCCC, for instance, have been employed in a number of studies, valued for their documentation of climate policy action across nations, over time, and according to specific guidelines, and perhaps offer one of the most comprehensive global datasets for adaptation tracking that we have. Nevertheless, NCs were not designed for tracking purposes per se, remain dominated by mitigation, are of varying quality in their documentation of adaptation, focus predominantly on the national level, and are insufficient for detailed analysis. The development of robust reporting systems to create a global adaptation inventory is therefore urgently needed, with the UNFCCC ideally suited to take leadership role in creating a standardized adaptation reporting platform.

These are critical times for adaptation, which has become firmly established in climate policy. Yet adaptation science remains the poor cousin of mitigation science, with significant differences in understanding and scientific development between the two. Adaptation tracking is one such area of divergence, where our ability to answer the question: are we adapting to climate change is limited by an absence of tools, datasets, and baseline research. Addressing these gaps should be a priority for future work, with the 4Cs proposed here providing a starting point for systematically examining adaptation progress.
